# Special Issue: From Natural Polyphenols to Synthetic Bioactive Analogues

**DOI:** 10.3390/molecules25122772

**Published:** 2020-06-16

**Authors:** Corrado Tringali

**Affiliations:** Department of Chemical Sciences, University of Catania, Viale A. Doria 6, 95125 Catania, Italy; ctringali@unict.it

In recent years, phenolic compounds from plant sources, commonly referred to as ‘plant polyphenols’, have been the subject of an impressive number of research studies, to a large extent focused on the healthy properties attributed to diet polyphenols, including antioxidant, anti-inflammatory, antineoplastic, antidiabetic, neuroprotective, and other biological activities. Additionally, phenolic compounds isolated from toxic plants and showing cytotoxic or antiproliferative activity have been intensively investigated in view of a possible exploitation of their anticancer properties. In parallel, many research groups have focused their work on obtaining synthetic or semisynthetic analogues of these molecules, with the aim of enhancing their biological activity and possibly improving their metabolic stability and bioavailability, as a first step towards the discovery of new chemotherapeutics agents. The preparation of libraries of analogues derived from natural polyphenols may also contribute to a better understanding of the molecular mechanisms of action of the most promising compounds through structure–activity relationship (SAR) studies. Finally, synthetic compounds inspired by a natural scaffold may also show new and unexpected biological properties.

Thus, this Special Issue aims to highlight recent results both in the field of natural polyphenols and in that of their synthetic bioactive analogues. It is composed of one review and four original articles, overall reporting results about the synthesis of antiproliferative bisphenol neolignans inspired by honokiol, a multicomponent synthesis of polyphenols as potential β-amyloid aggregation inhibitors, polyphenols from *Tamarix ramosissima* and *Melanoleuca styphelioides* as potential antioxidant, antimicrobial or anti-inflammatory agents, and a review article on aza- and azo-stilbenes as bioisosteric analogs of resveratrol.

Cardullo et al. [1] report the synthesis of a library of bisphenol neolignans inspired by honokiol, a natural polyphenol showing a variety of biological properties, including antitumor activity. The natural lead was subjected to simple chemical modifications to obtain a first group of derivatives. To obtain further neolignans with a different substitution pattern to honokiol, the Suzuki–Miyaura reaction was employed. These compounds and the natural lead were subjected to antiproliferative assay towards HCT-116, HT-29, and PC3 tumor cell lines. Six neolignans show GI_50_ values lower than those of honokiol towards all cell lines. Three compounds showed GI_50_ in the range of 3.6–19.1 µM, in some cases lower than those of the anticancer drug 5-fluorouracil. Flow cytometry experiments showed that the antiproliferative activity is mainly due to an apoptotic process.

The paper by Galante et al. [2] describes an example of application of the Ugi multicomponent reaction to the combinatorial assembly of artificial, yet “natural-like”, polyphenols. The authors used a “natural fragment-based approach” to the combinatorial synthesis of polyphenolic molecules. Starting from small phenolic building blocks, they obtained a series of artificial polyphenols, which were evaluated as inhibitors of β-amyloid protein aggregation and potential anti-Alzheimer agents The biochemical assays highlighted the importance of the key pharmacophores in the synthesized compounds. As final result, a lead for inhibition of aggregation of truncated protein AβpE3-42 was selected.

A further contribution by Ren et al. [3] is focused on polyphenols from *Tamarix ramosissima* bark, to determine their potential antioxidant and antimicrobial activities. A total of 13 polyphenolic compounds were identified by UPLC-MS analysis. Hispidulin and cirsimaritin, active ingredients of traditional Chinese herbs, were identified for the first time in a *Tamarix* sp. The main constituents of bark extract are isorhamnetin (36.91 μg/mg extract), hispidulin (28.79 μg/mg) and cirsimaritin (13.35 μg/mg). The antioxidant activity of the bark extract was evaluated through DPPH, ABTS, the superoxide anion and hydroxy radical scavenging, ferric reducing power and FRAP. Promising results were obtained for DPPH (IC_50_ value of 117.05 μg/mL), hydroxyl radical scavenging (151.57 μg/mL) and reducing power (EC_50_ of 93.77 μg/mL). The *T. ramosissima* bark extract showed antibacterial activity against foodborne pathogens. *Listeria monocytogenes* was the most sensitive microorganism with the lowest minimum inhibitory concentration (MIC) value of 5 mg/mL and minimum bactericidal concentration (MBC) value of 10 mg/mL, followed by *Shigella castellani* and *Staphylococcus aureus* among the tested bacteria.

Albouchi et al. [4] present a study on *Melaleuca styphelioides*, known as the prickly-leaf tea tree. The authors characterized the polyphenols extracted from the leaves and determined their potential antioxidant and anti-inflammatory activity. LC/MS-MS was used to identify and quantify the phenolic compounds. An assessment of the radical scavenging activity of all extracts was performed using DPPH, ABTS^+^ and FRAP assays. The anti-inflammatory activity was determined on interferon gamma (IFN-γ)/histamine (H)-stimulated human NCTC 2544 keratinocytes by Western blot and RT-PCR. The methanolic extract presented the highest concentration of phenolics. The main constituents were quercetin, gallic acid and ellagic acid. DPPH, ABTS^+^, and FRAP assays showed that methanolic extract exhibits strong concentration-dependent antioxidant activity. IFN-γ/H treatment of human NCTC 2544 keratinocytes induced the secretion of high levels of the pro-inflammatory mediator inter-cellular adhesion molecule-1 (ICAM-1), nitric oxide synthase (iNOS), cyclooxygenase-2 (COX-2), and nuclear factor kappa B (NF-κB), which were inhibited by the extract. In conclusion, the extract of *Melaleuca styphelioides* can be proposed as a useful treatment for inflammatory skin diseases.

Finally, this Special Issue includes a review article by Lizard et al. [5], devoted to aza- and azo-stilbenes as bioisosteric analogs of resveratrol. Stilbenoid polyphenols are well known for their promising biological properties. However, their moderate bio-availabilities, especially for trans-resveratrol, prompted a number of researchers to optimize their properties by synthesizing innovative resveratrol analogs. The review is focused on isosteric resveratrol analogs, namely aza-stilbenes and azo-stilbenes, in which the central double bond is replaced with C=N or N=N bonds, respectively. The biological activities of some of these molecules are reported in view of their potential therapeutic applications. In some cases, structure–activity relationships are discussed.

We expect that this Special Issue will promote interest in the search for bioactive polyphenols as potential therapeutic agents.
